# Effect of an individualized versus standard blood pressure management during mechanical thrombectomy for anterior ischemic stroke: the DETERMINE randomized controlled trial

**DOI:** 10.1186/s13063-022-06538-9

**Published:** 2022-07-26

**Authors:** Benjamin Maïer, Benjamin Gory, Russell Chabanne, Benoît Tavernier, Baptiste Balanca, Gérard Audibert, Laurie-Anne Thion, Morgan Le Guen, Thomas Geeraerts, Lionel Calviere, Vincent Degos, Bertrand Lapergue, Sebastien Richard, Azeddine Djarallah, Ornellia Mophawe, Perrine Boursin, Chloé Le Cossec, Raphael Blanc, Michel Piotin, Mikael Mazighi, Etienne Gayat, on behalf of the DETERMINE Investigators, Benjamin Maïer, Simon Escalard, François Delvoye, Solène Hebert, Hocine Redjem, Stanislas Smajda, Jean-Philippe Desilles, Raphael Blanc, Michel Piotin, Mikael Mazighi, Amélie Yavchitz, Chloé Le Cossec, Ornellia Mophawe, Azedine Djarallah, Perrine Boursin, Laurie-Anne Thion, Abdenour Amarouche, Anoushee Shaffii, Audrey Fogang, Aurore Marcou, Elisabeth Ferri, Fanny Le Garrec, Ines Da Costa, Jean-Marie Moures, Jean-Michel Devys, Malika Omarjee, Marie-Claude Dubois, Marie-Claire Nghe-Mann, Matthieu Dorison, Mélanie Sénéchal, Pascal Le Bigot, Nouria Belhadj-Tahar, Severine Gras, Simon Clariot, Stéphane Merat, Sylvie Froucht-Hirsch, Yasmine Ait Yahia, Pierre Seners, Candice Sabben, Bertrand Lapergue, Morgan Le Guen, Julien Rousset, Thomas Geeraerts, Christophe Cognard, Jean-Marc Olivot, Lionel Calviere, Maxime Pommier, Edouard Naboulsi, Marc Begard, Camille Boissy, Thibaud Cammas, Bernard Cosserant, Romain Grobost, Adrien Guyot, Katia Levrier, Pierre-Antoine Pioche, Ricardo Moreno, Abderahim Zerroug, Elie Lteif, Emmanuel Chabert, Anna Ferrier, Aurélie Masgrau, Baptiste Balanca, Lionel Bapteste, Baptiste Bouchier, Claudio Di Roio, Charles-Antoine Lak, Anisoara Gemanar, Romain Carrillon, Carole Bodonian, Benjamin Gory, Serge Bracard, René Anxionnat, Marc Braun, Anne-Laure Derelle, Liang Liao, François Zhu, Emmanuelle Schmitt, Sophie Planel, Sébastien Richard, Lisa Humbertjean, Gioia Mione, Jean-Christophe Lacour, Marian Douarinou, Gérard Audibert, Marcela Voicu, Lionel Alb, Marie Reitter, Madalina Brezeanu, Agnès Masson, Adriana Tabarna, Iona Podar, Pauline Bourst, Valérie Georges, Sarah Guy, Fatiha Bechiri, Benoît Tavernier, Gabriela Julean, Pierre Boussemart, Sidi Hamza Roudies, Dominique Envain, Pierre Appourchaux, Julien Martin, Victor Lestrade, Lucie Della Schiava, Nicolas Bricout, Erine Prévost, Julie Bellet, Etienne Gayat, Mikael Mazighi, Vincent Degos, Dupont Julie, Frédéric Clarençon

**Affiliations:** 1 Interventional Neuroradiology Department, Hôpital Fondation Adolphe de Rothschild, 29 rue Manin, 75019 Paris, France; 2 Université Paris-Cité, Paris, France; 3 Diagnostic and Therapeutic Neuroradiology Department, CHRU-Nancy, Université de Lorraine, INSERM U124, Nancy, France; 4 Department of Anesthesia, Critical Care and Peri-Operative Medicine, Centre Hospitalier Universitaire de Clermont-Ferrand, Clermont-Ferrand, France; 5 Department of Anesthesia and Critical Care, University Hospital, Lille, F-59000 Lille, France; 6 Université Lille, ULR 2694 – METRICS, F-59000 Lille, France; 7 Department of Neurological Anesthesiology and Intensive Care, Hospices Civils de Lyon, Hôpital Pierre Wertheimer, Groupement Hospitalier Est, 59 Boulevard Pinel, 69500, Bron, Lyon, France; 8 Lyon’s Neuroscience Research Center, INSERM U1028/CNRS UMR 5292, Lyon 1 University, Lyon, France; 9 Anesthesiology Department, CHRU Nancy, Nancy, France; 10 Anesthesiology Department, Hôpital fondation A. de Rothschild, Paris, France; 11 Anesthesiology Department, Foch Hospital, Suresnes, France; 12 Anesthesiology and Critical Care department, University Hospital of Toulouse, University Toulouse 3-Paul Sabatier, Toulouse, France; 13 Neurology Department, University Hospital of Toulouse, Toulouse, France; 14 Department of Anesthesia, Critical Care and Peri-Operative Medicine, APHP, Sorbonne Université, Hôpital Pitié-Salpêtrière, Paris, France; 15 INSERM UMR 1141, Paris, France; 16 Neurology Department, Stroke Unit, Foch Hospital, Suresnes, France; 17 Neurology Department, CHRU-Nancy, Université de Lorraine, Nancy, France; 18 Clinical Research Unit, Hôpital fondation A. de Rothschild, Paris, France; 19 Anesthesiology Department, Hôpital Lariboisière, Paris, France

**Keywords:** Stroke, Blood pressure, Thrombectomy, Disability, Mean arterial pressure, Circulation, Randomized controlled trial

## Abstract

**Background:**

Hypotension and blood pressure (BP) variability during endovascular therapy (EVT) for acute ischemic stroke (AIS) due to an anterior large vessel occlusion (LVO) is associated with worse outcomes. However, the optimal BP threshold during EVT is still unknown given the lack of randomized controlled evidence. We designed the DETERMINE trial to assess whether an individualized BP management during EVT could achieve better functional outcomes compared to a standard BP management.

**Methods:**

The DETERMINE trial is a multicenter, prospective, randomized, controlled, open-label, blinded endpoint clinical trial (PROBE design). AIS patients with a proximal anterior LVO are randomly assigned, in a 1:1 ratio, to an experimental arm in which mean arterial pressure (MAP) is maintained within 10% of the first MAP measured before EVT, or a control arm in which systolic BP (SBP) is maintained within 140–180 mm Hg until reperfusion is achieved or artery closure in case of EVT failure. The primary outcome is the rate of favorable functional outcomes, defined by a modified Rankin Scale (mRS) between 0 and 2 at 90 days. Secondary outcomes include excellent outcome and ordinal analysis of the mRS at 90 days, early neurological improvement at 24 h (National Institutes of Health Stroke Scale), final infarct volume, symptomatic intracranial hemorrhage rates, and all-cause mortality at 90 days. Overall, 432 patients will be included.

**Discussion:**

DETERMINE will assess the clinical relevance of an individualized BP management before reperfusion compared to the one size fits all approach currently recommended by international guidelines.

**Trial registration:**

ClinicalTrials.gov, NCT04352296. Registered on 20th April 2020.

**Supplementary Information:**

The online version contains supplementary material available at 10.1186/s13063-022-06538-9.

## Background

Endovascular therapy (EVT) is now the reference treatment of acute ischemic stroke (AIS) due to an anterior large vessel occlusion (LVO) [1, 2]. Despite reperfusion rates commonly exceeding 90% at the end of procedure [3], clinical outcomes at 90 days remain unsatisfactory with half of reperfused patients experiencing unfavorable outcomes [4]. Among factors likely responsible for this result, recent studies have shed light on the pivotal role of blood pressure (BP) variability and hypotension during EVT [5–14]. Several studies have shown that hypotension and BP variability during EVT were associated with worse functional outcome at 90 days [5–7, 10, 12], as well as larger infarct volume [6]. Hypotension during EVT is frequent, mainly explained by anesthetics drugs given to perform EVT under conscious sedation (CS) or general anesthesia (GA) [6]. In this context, a recent analysis of individual patient data from 3 randomized controlled trials (RCT), initially designed to evaluate the best sedation modality for EVT, concluded that mean arterial pressure (MAP) could be a modifiable therapeutic target to prevent or reduce poor functional outcomes and should be maintained within narrow limits [7]. In line with this work, a recent study highlighted the deleterious impact of hypotension during EVT, with a linear association between hypotension and functional outcomes at 90 days [14]. In this study, the odds ratio for poor functional outcome of only 10 min under 90% of the baseline mean arterial pressure (i.e., the first MAP measured in the angio suit, before EVT) was 1.11 (1.02–1.21) [14]. Interestingly, the association between the depth of hypotension and worse functional outcomes was similar for either CS and GA, highlighting the deleterious effect of the hemodynamic management during EVT [14]. The efficacy of an individualized BP management has already been proven for general surgery, using diluted norepinephrine in order to stabilize BP within narrow limits [15]. Applied to EVT, this individualized management seems attractive as the BP target to reach before reperfusion could differ among patients, a hypothesis that has to be studied in dedicated RCT [5, 8, 9, 16]. Current guidelines for BP management during EVT only suggest maintaining BP below 180/105 mm Hg during the procedure, without addressing the potential deleterious effect of hypotension, nor BP variability [1, 2, 17]. On the other hand, the Society for Neuroscience in Anesthesiology and Critical Care (SNACC) addressed the issue of hypotension during EVT and recommended that systolic BP (SBP) should be maintained >140 mm Hg (class IIa, level of evidence B) [18]. These guidelines were primarily derived from data evaluating the impact of BP in patients treated only with intravenous thrombolysis (with and without LVO), given the lack of RCT in the EVT era.

With this as a background, the DETERMINE trial aims to assess the efficacy on favorable functional outcomes at 90 days of an individualized BP management during EVT, by maintaining MAP within 10% of the first MAP measured before EVT, compared to a standard BP management during EVT (SBP within 140–180 mm Hg, DBP < 105 mm Hg).

## Methods/design

### Design

**Fig. 1:**
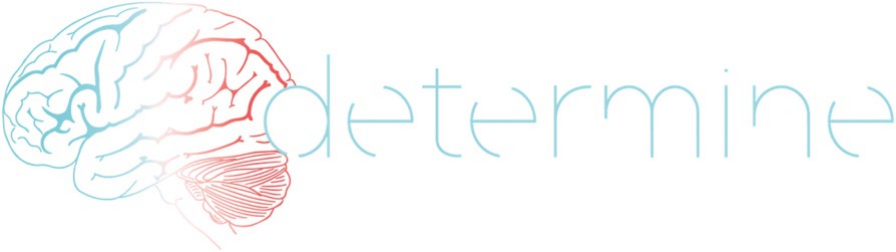
DETERMINE

DETERMINE is an academic, multicenter, prospective, randomized, open-label, with blinded endpoint assessment clinical trial (PROBE) (Fig. 1). This trial, funded by the French Health Ministry, sponsored by the Hôpital Fondation A. de Rothschild, is currently conducted in 9 comprehensive stroke centers in France. The first patient was included in March 2021.

### Inclusion and exclusion criteria

#### Inclusion criteria

1. Adult patients (≥18 years)
2. AIS due to an anterior LVO: first and second segments of the middle cerebral artery, first segment of the anterior cerebral artery, intracranial internal carotid artery, tandem occlusions (association of an extracranial occlusion of the cervical internal carotid artery with a proximal anterior LVO)
3. Indication of EVT under CS or GA, within the first 6 h after symptoms onset according to current guidelines [1, 2], or within 24 h according to neuroradiological criteria previously published [1, 2]
4. Affiliation to a social security scheme

#### Exclusion criteria

1. Contraindication or no indication for EVT
2. Intubation or GA induction before randomization
3. Intra-hospital AIS due to an anterior LVO or due to a medical or surgical procedure (interventional cardiology, cardiac or vascular surgery) or any post-surgery AIS
4. AIS due to a posterior LVO (vertebral artery, basilar artery or posterior cerebral artery occlusions)
5. Significant pre-stroke functional disability (modified Rankin Scale >2 at randomization)
6. Contraindication to iodinated contrast products
7. Patients benefiting from legal protection measures
8. Pregnant or breastfeeding woman

#### Secondary exclusion criteria

Failure to measure and define the reference MAP within a timeframe compatible with the timely emergency management of the patient: difference >10 mmHg between the first 2 MAP measured non-invasively at 1 min interval, and a third MAP >10 mm Hg of the mean of the first 2 MAP (see below).

### Randomization

Randomization is performed during the installation of the patient in the angio suit (which takes approximately 10 min). To ensure a centralized and real-time procedure, online randomization is performed via an eCRF using Clinfile software (https://determine.clinfile.com). The server allocates the treatment group on the basis of a minimization process to balance in a 1:1 ratio the 2 groups, stratified by age (< 70 versus ≥70 years), sedation modality (GA versus CS), baseline NIHSS (< 17 versus ≥17), intravenous thrombolysis (yes versus no), and the center in which the patient is included.

### Intervention

#### Patient installation, definition of the reference MAP, and BP monitoring

A BP monitor cuff is placed on the arm, contralateral to the diluted norepinephrine infusion, adapted to the patient’s morphology. The reference MAP is defined by the mean of the first 2 MAP measured at 1-min intervals, if these values do not differ by 10 mmHg. If these first 2 MAP differ by more than 10 mmHg, a third MAP is measured after resolving a potential technical issue. If the third MAP differs by less than 10 mmHg of the first mean, the reference MAP is the mean of the 3 values. Exceptionally, the third measure exceeds 10 mmHg of the first mean, and randomization is not performed to prevent any delay for EVT. The reference MAP is automatically generated by the randomization website to prevent any calculation error and avoid any delay in the start of EVT.

Due to the absence of validated or recommended protocol for BP measurement during EVT, BP is measured non-invasively, every 2.5 min, as this is the current standard practice in France. If an arterial line is placed before EVT, values are collected for exploratory purposes, but only non-invasive measures are used for BP management.

#### Experimental arm

**Fig. 2:**
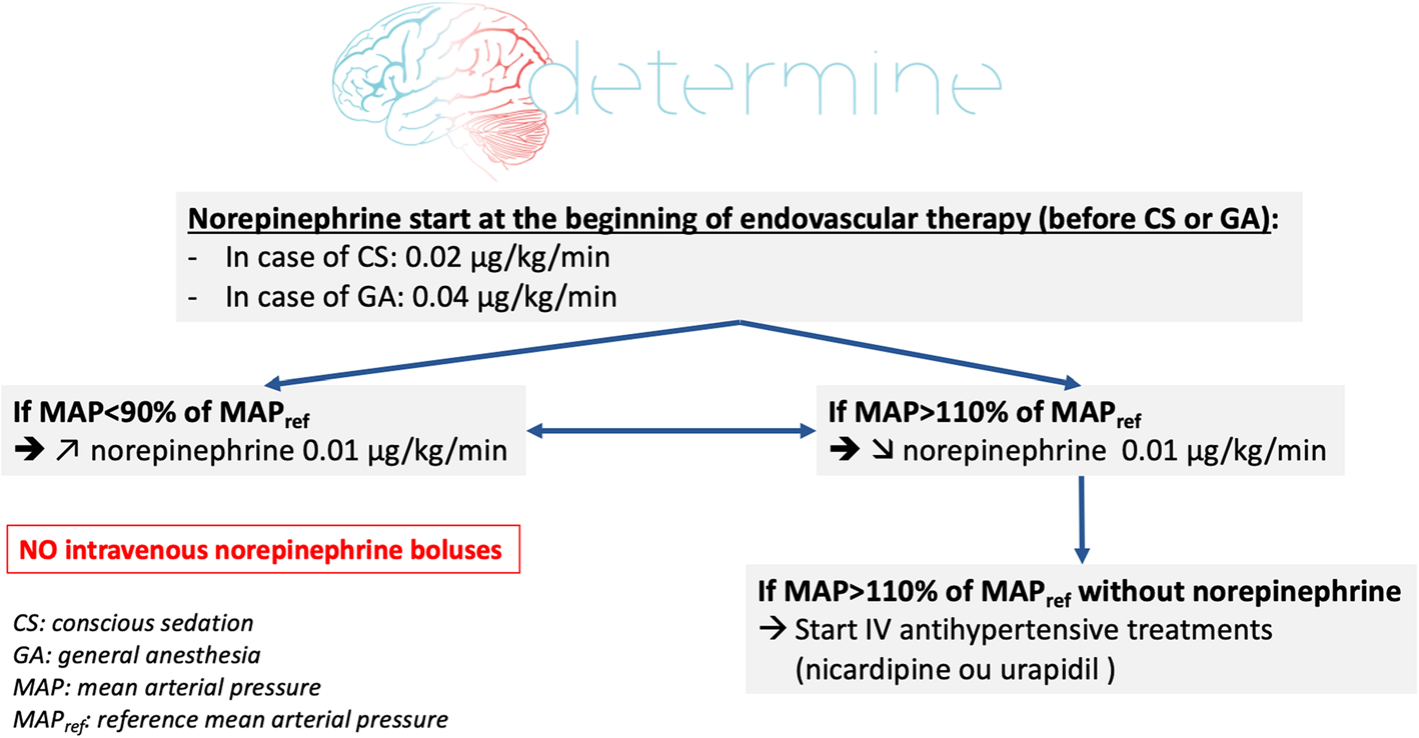
Hemodynamic protocol

The objective is to maintain MAP within 10% of the reference MAP during EVT. This BP target is achieved using intravenous diluted norepinephrine (5 or 10 μg/ml) to prevent and treat hypotension (< 10% of the reference MAP) and intravenous nicardipine or urapidil for hypertension (> 10% of the reference MAP). Norepinephrine is prepared in a dedicated syringe, administered on a dedicated peripheral venous line or on a 3-way venous extender and started systematically before any GA induction (0.04 μg/kg/min) or CS initiation (0.02 μg/kg/min) to avoid hypotension. Norepinephrine administration is continuous, without boluses, adapted to the patient’s weight (Additional file 2). The hemodynamic protocol is presented in Fig. 2.

In case of hypertension (MAP > 110% of the reference MAP), a decrease of norepinephrine doses is achieved (Fig. 2 and Additional file 2). In case of the persistence of hypertension and discontinuation of norepinephrine, hypertension is treated by currently validated drugs: nicardipine, urapidil.

#### Control arm

Blood pressure management is based on current guidelines, primarily oriented towards the treatment of hypertension (SBP > 180 mmHg and DBP > 105 mmHg) [17]. Hypertension is treated with validated drugs (intravenous nicardipine or urapidil). As recommended by the SNACC, hypotension is only treated for SBP < 140 mmHg [18]. Due to the absence of validated or recommended protocol to treat hypotension in this context, diluted norepinephrine, as well as ephedrine (3mg/ml) or phenylephrine (50μg/ml) can be used. No pharmacological intervention is undertaken if BP is within these targets.

Intervention is complete at the time of reperfusion in case of successful EVT (i.e., modified Treatment in Cerebral Ischemia -mTICI- between 2b and 3) or the placement of the femoral artery closure device in case of EVT failure (i.e., mTICI < 2b). As most of patients after EVT are subsequently referred to stroke units, where diluted norepinephrine infusion cannot be used, discontinuation of this treatment is performed in the angio suit at the end of intervention.

### Procedure care and follow-up

**Fig. 3:**
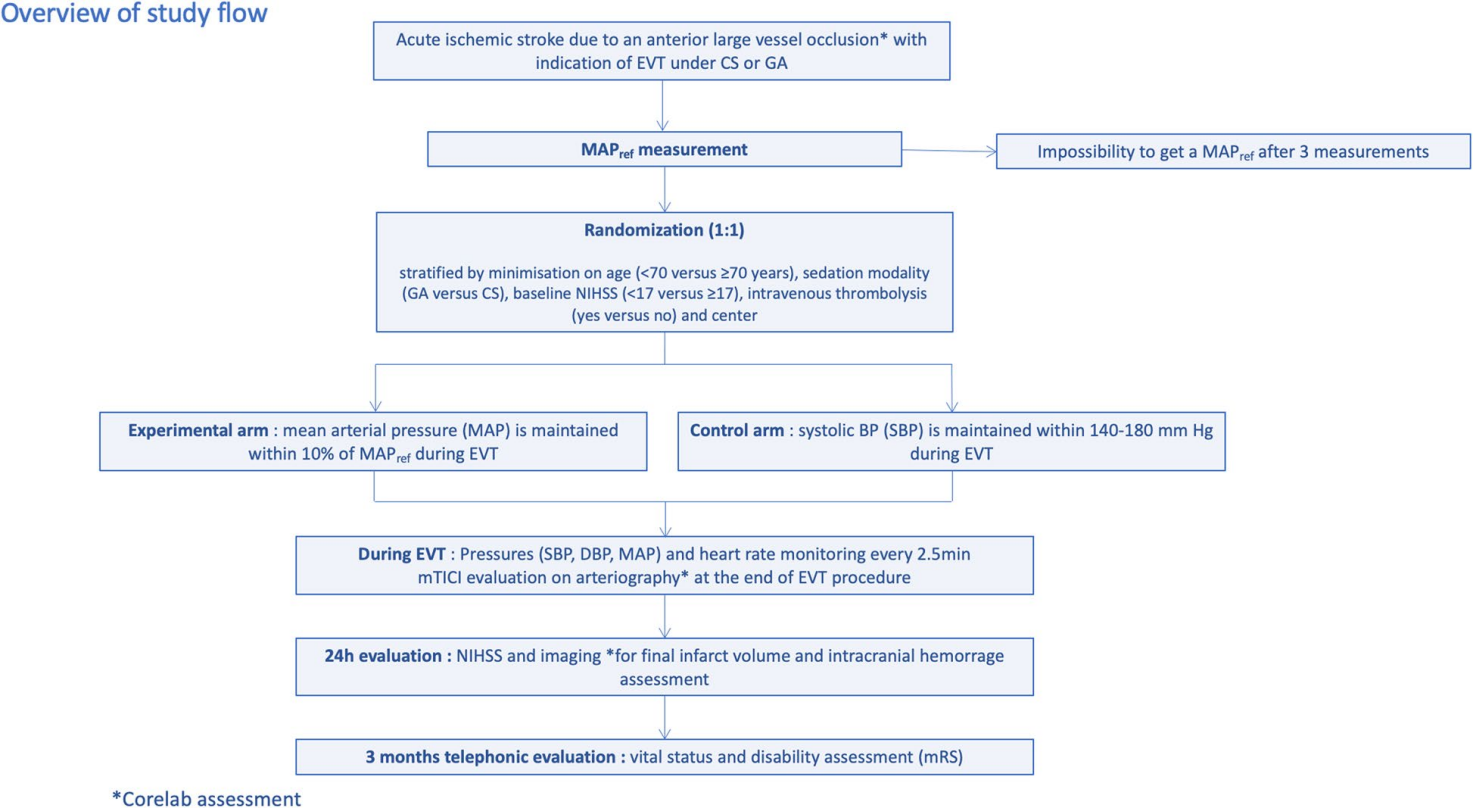
Overview of the DETERMINE trial

**Fig. 4:**
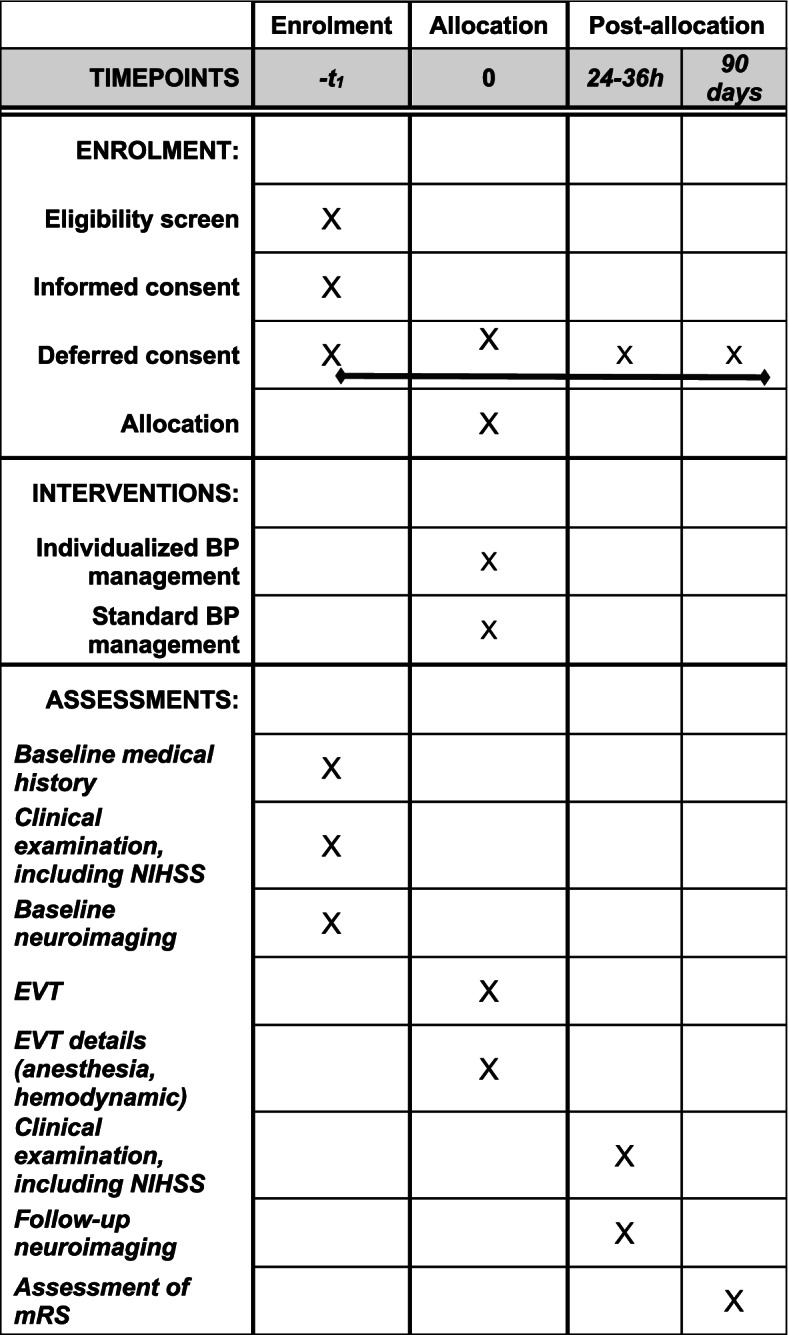
Overview of the DETERMINE trial

EVT are performed by senior neuroradiologists in dedicated bi-plan angiography suits, using last-generation devices (aspiration, stent retrievers). Sedation, as well as BP management, is performed by senior anesthesiologists. Sedation modality is discussed collegially between the interventional neuroradiologist, anesthesiologist, and the neurologist in charge at the beginning and during EVT (see Additional file 3 for the sedation protocol). All treatments in the acute phase (antithrombotic drugs, technical intervention strategies used for EVT) and the management of post-AIS complications (neurological, respiratory) are in accordance with current guidelines [1]. At 24h, patients will undergo a NIHSS assessment by a certified neurologist, unaware of the randomization group. A non-contrast brain computed tomography or a brain magnetic resonance imaging (MRI) will also be performed at 24h. Functional outcomes are assessed at 90 days, as explained below. An overview of the DETERMINE trial is provided in Fig. 3 and Fig. 4.

### Outcomes

#### Primary endpoint

Favorable functional outcome, defined by a modified Rankin Scale (mRS) between 0 and 2 at 90 days (± 15 days), assessed by certified neurologists or research nurses, blinded to the intervention.

#### Secondary endpoints

1. Distribution of the mRS at 90 days (shift analysis)
2. Excellent functional outcome, defined by a mRS between 0 and 1 at 90 days
3. Early neurological improvement, defined by a reduction of 8 points or more on the NIHSS or NIHSS 0-1 24 h after EVT
4. Successful reperfusion, defined as a modified Treatment In Cerebral Infarctions 2b-3
5. Final infarct volume 24 h after EVT on control cerebral imaging
6. Frequency and length of hospital stays
  - Duration of initial hospital stay (days)
  - Number of re-hospitalizations within 90 days
  - Cumulative length of hospitalization during the 90 days follow-up
7. Description of per procedural BP in each group:
  - Percentage of patients with at least one per-procedural hypotension, total number of per-procedural hypotension, mean duration of hypotension (MAP < 90% of the reference MAP for the experimental group, SBP < 140 mmHg for the control group)
  - Percentage of patients with at least one per-procedural hypertension, total number of per-procedural hypertension, mean duration of hypertension (MAP > 110% of the reference MAP for the experimental group, SBP > 180 and DBP > 105 mmHg for the control group)
  - Percentage of patients with at least one per-procedural bradycardia, total number of per-procedural bradycardia, mean duration of bradycardia (< 40/min)
  - BP variability, defined by the coefficient of variation
  - In the experimental group, time spent within the experimental target (± 10% of the reference MAP) during EVT (in minutes and in % of the total EVT duration)

#### Safety endpoints

1. Symptomatic intracranial hemorrhage according to the ECASS III definition [19]
2. Symptomatic intracranial hemorrhage 24 h after MT according to the SITSMOST definition [20]
3. All-cause mortality at 90 days

### Core-lab analysis

Baseline and control cerebral imaging, as well as angiograms, are deidentified so that all neuroimaging secondary endpoints are assessed by a central core-lab, also blinded to the intervention.

### Informed consent

After verification of the inclusion and exclusion criteria, the interventional neuroradiologist and neurologist explain the objectives and the course of the study to the patient using an information form and seek for his/her consent. However, given the patient’s clinical condition (high probability that he/she is unable to give consent) and the urgency of his/her care, the consent of a family member or trusted person present is sought, without delaying the patient's care. As an exception, if the patient is unable to express consent and no relative is present, the physician may include the patient according to the procedure for inclusion in an immediate life-threatening emergency. As soon as possible and when the patient has regained a sufficient state of consciousness, his or her consent to the continuation of the research and to the processing of the information collected is sought. If the patient has not regained a sufficient state of consciousness, the consent of a close relative (family member or trusted person) to continue the research is sought as soon as possible.

### Data safety monitoring board (DSMB)

The DSMB is composed of one neurologist, one anesthesiologist, and one methodologist, not participating in the study and not affiliated with the sponsor. The DSMB will review on a regular basis the efficacy and safety data, to make recommendations to the principal investigator and the steering committee to stop the trial based on the occurrence of adverse events and serious adverse events. The DSMB charter can be communicated upon request to the sponsor.

### Adverse and serious adverse events

All adverse events and serious adverse events (SAE) are systematically reported in the eCRF (specific notification form) by the investigators. For each adverse event, the investigator evaluates its intensity, its severity, and the causal link with the intervention or with other possible treatments.

In addition to SAEs, the investigator promptly notifies the following events that have been identified as requiring monitoring throughout the research:

- Severe hemodynamic variation during EVT: Incidence of severe hypotension (SBP < 90 mmHg or MAP < 55 mmHg for more than 10 min for both groups)
- Incidence of severe hypertension (SBP > 200 mmHg or DBP > 120 mmHg for more than 10 min for the control group or MAP increase of more than 40% for more than 10 min for the experimental group)
- Incidence of severe bradycardia (any extreme bradycardia requiring emergency treatment with atropine or adrenaline)
- Symptomatic hemorrhagic transformation within the first 24 h: appearance of extravascular and intracranial blood on follow-up brain imaging performed between 24 and 36 h after EVT, associated with clinical deterioration defined by an increase of 4 or more points in the NIHSS score or resulting in death of the patient, for which the primary cause is attributable to hemorrhage (ECASS III definition)
- Parenchymal hematoma within the first 24 h, type PH2
- Symptomatic cerebral edema during hospitalization requiring hemicraniectomy

For each SAE, the vigilance officer assesses the seriousness and the link with the intervention and, if necessary, judges whether it is expected or unexpected. The vigilance officer declares to the authorities (Agence Nationale de Sécurité du Médicament et des produits de santé) any suspected or unexpected serious adverse reaction.

### Data management

Data are recorded in an electronic case report form (e-CRF), developed using Clinfile©. All data are entered in the e-CRF by the study research team at each center. Every reasonable effort should be made to complete data entry as soon as possible (maximum within 2 weeks) from data collection. The principal investigator/designee is responsible for the accuracy and completeness of recorded data. The e-CRF was created, tested, and validated before the start of data entry. The data necessary for monitoring the primary and secondary endpoints are identified and managed at regular intervals throughout the trial. Data are monitored using predefined data management rules and queries are automatically edited. Finally, an overall automated monitoring is performed by the data manager at the end of the data entry. In case of discrepancies, queries are edited to resolve the problems encountered. After validation, the database will be frozen and exported to the statistical software package for analysis.

### Ethical considerations

The study has obtained research ethics approval from the Comité de Protection des Personnes Ouest V on September 29, 2020, and by the Agence Nationale de Sécurité du Médicament et des produits de santé (ANSM) on July 30, 2020. Any modifications to the protocol which may impact on the conduct of the study, potential benefit of the patient or may affect patient safety, including changes of study objectives, study design, patient population, sample sizes, study procedures, or significant administrative aspects, require a formal amendment to the protocol. Such amendments are approved by the Comité de Protection des Personnes (CPP) prior to implementation and notified to all investigators by email with all the updated study documents.

### Organization

- *The steering committee* consists of principal investigators of the participating centers (anesthesiologists, neurologists and interventional neuroradiologists) and two methodologists initially implicated in the study design. The steering committee met before the first enrollment, to discuss the study design and the practical implementation at each center and meets annually to discuss study progress and amendments.
- *The scientific committee* consists of the principal investigator of the study, one neurologist and one anesthesiologist, directly implicated in the study design and the application for the academic funding of the study.
- *The executive committee* consists of the principal investigator of the study and two local principal investigators at each center (one anesthesiologist and one neurologist or interventional neuroradiologist). They provide frequent feedbacks on difficulties regarding the implementation of the trial, completion of the eCRF, or any relevant issue that needs to be discussed with the steering committee.

The investigators and collaborators of the DETERMINE trial is given in Additional file 1.

### Sample size

Expected rate of poor functional outcomes at 90 days in the control arm is 54% [10]. Based on recently published observational data [5], we estimated the relative risk of intervention to be around 0.7. Using a more conservative value, an alpha risk of 5%, and an interim efficacy analysis after 50% of inclusions based on an O’Brien and Fleming risk expenditure procedure, inclusion of 432 randomized patients would demonstrate a hazard ratio of 0.75 with 80% power.

### Statistical analysis

Principal analysis will be conducted on the intention-to-treat (ITT) population. All randomized patients will be analyzed according to their randomization arm. Any missing data on the primary endpoint will be imputed using a multiple imputation procedure.

The primary endpoint is favorable functional outcomes at 90 days, defined as a modified Rankin score (mRS) between 0 and 2. The rate of patients with a favorable functional outcome at 90 days will be compared between the 2 groups using a mixed-effect logistic regression model, adjusted for the variables considered in the randomization, namely age (< 70 vs. ≥ 70 years), type of anesthesia (general vs. conscious sedation) NIHSS score at inclusion (< 17 vs. ≥ 17), and IV thrombolysis (yes vs. no) as fixed effects, and center as a random effect. The adjusted odds ratio (OR) will be calculated from this model. The secondary endpoints corresponding to binary variables (criterion 2, 3, 4, 5, 6, 8) will also be analyzed using a mixed-effects logistic regression model, adjusted for the variables considered in the randomization. The ordinal analysis of the modified Rankin score at 90 days (criterion 1) will be performed using a mixed ordinal logistic regression model, adjusted on the variables considered in the randomization. The analysis of the number of re-hospitalizations within 90 days (criterion 9b) will be performed using a mixed effects Poisson regression (quasi fish or negative binomial), adjusted on the variables considered in the randomization. Secondary endpoints corresponding to quantitative variables (criterion 7, 9a, 9c) will be analyzed using mixed-effects linear models, adjusted for the variables considered in the randomization. In case of skewed distribution of these parameters, lognormal or gamma regression may be used. Hemodynamic variations (criterion 10) during EVT and the fraction of operating time spent in the target (± 10% of the first MAP measured in the experimental group (criterion 11) will be described as numbers and percentages for qualitative variables, mean, standard deviation, median and range for quantitative variables.

An interim efficacy analysis will be performed after 50% of inclusions. The alpha risk thresholds to be used in the interim and final analysis are 0.003 and 0.049, respectively. The DSMB, the sponsor, and the principal investigator will have access to the results of the interim analysis.

Efficacy as defined for the primary outcome will be analyzed by the following subgroups: age (< 70 years, ≥ 70 years), time to management ( <180 min, 180–360 min, > 360 min), history of hypertension (yes vs no); NIHSS at inclusion (< 17 vs ≥17), sex (female vs male), type of sedation (conscious sedation vs general anesthesia), IV thrombolysis (yes vs no), occlusion site ( M1-M2 , ICA termination , tandem), inclusion systolic blood pressure (first measurement in the operating room < 140, 140–179, ≥ 180 mmHg), inclusion mean blood pressure (first measurement in the operating room < 90, 90–110, > 110 mmHg), and center. A statistical analysis plan of the DETERMINE trial is provided in the Additional file 4.

## Discussion

To date, only one RCT assessed the efficacy and safety of BP control after successful EVT (BP TARGET) (i.e., after reperfusion) [21] and randomized controlled evidence regarding BP control during EVT (i.e., before reperfusion) are still lacking, despite several recent observational data highlighting the deleterious effect of hypotension during EVT [5–8, 10, 14].

Recent observational studies have shown the potential value of an individualized BP management before reperfusion, as the impact of hypotension during EVT differed according to the collateral status or the anatomy of the Circle of Willis (presence or absence of a posterior communicating artery). Nevertheless, recent European guidelines recommend a one size fits all approach with a unique BP target during EVT (< 180/105 mm Hg), mainly explained by the absence of RCT in the EVT era.

In contrast to the INPRESS trial for general surgery, in which SBP was used as the primary hemodynamic parameter, the DETERMINE trial uses MAP [15]. This choice was based on several factors. First, numerous studies have shown the association between hypotension defined by different MAP thresholds with worse functional outcomes at 90 days [5, 7, 8, 13, 14, 22]. Second, we previously applied the INPRESS criteria to EVT, using MAP to describe BP drops and hypotension time during EVT and found strong associations between lower MAP value and functional outcomes [14]. Finally, MAP is a neurologically relevant hemodynamic parameter due to its association with cerebral perfusion pressure and intracranial pressure [5, 7, 11].

### Other trials

The INDIVIDUATE (Individualized BP management during Endovascular Stroke Treatment) trial (NCT04578288) is also evaluating the efficacy of an individualized BP strategy before reperfusion of AIS due to an anterior LVO [23]. INDIVIDUATE is a single-center, parallel-group, open-label, randomized controlled trial with blinded endpoint evaluation and is planning to include 250 patients. The experimental arm is defined by the maintenance of baseline SBP within 10 mm Hg during EVT. As DETERMINE, the control arm of INDIVIDUATE is the maintenance of SBP between 140 and 180 mm Hg. The primary outcome is the functional outcome at 90 days using the mRS (0-2 versus 3-6). Interestingly, these trials share the same objective of proving the value of an individualized BP management during EVT.

The IDEAL (BP Management during EVT for AIS) trial (NCT04749251) is a pilot randomized clinical trial, which will evaluate the feasibility of an individualized BP management (maintenance of MAP within 10% of the first MAP measured) compared to the standard of care (MAP within 70–90 mm Hg). The IDEAL trial is also a single-center RCT and will include 60 patients treated with EVT for an anterior LVO under GA.

The MASTERSTROKE trial (Protocol for the Management of SBP during thrombectomy by endovascular route for AIS randomized clinical trial) is a multicentric RCT and will assess the efficacy of induced hypertension in AIS patients treated by EVT under GA for an anterior LVO [24]. MASTERTROKE has therefore a different design, as the DETERMINE and INDIVIDUATE trial do not aim to evaluate the efficacy of induced hypertension but evaluate the efficacy of an individualized BP management defined by patient-specific BP targets before reperfusion.

In line with most RCTs regarding BP control in the setting of AIS, it is likely that a major limitation of the DETERMINE trial will be the efficacy of BP control, especially for the experimental group given the narrow BP limits defined by our protocol (i.e., 10% within the first MAP measured before EVT). However, the participating centers belong to the Endovascular Treatment in Ischemic Stroke (ETIS, NCT03776877) registry and are used to manage EVT-treated patients with dedicated anesthesiologic teams, well aware of the relevance of hemodynamic control during EVT. Finally, thanks to wide and pragmatic inclusion criteria (basically, almost every EVT for proximal anterior LVO), DETERMINE will give new insights into the effect of hemodynamic control before reperfusion.

## Conclusions

No previous head-to-head randomized trials have directly compared the value of an individualized BP management during EVT. The DETERMINE trial is a multicenter, randomized, open-label, with blinded endpoint assessment clinical trial which aims to assess whether maintaining MAP within narrow limits during EVT (± 10% of baseline MAP, individualized management) can improve functional outcomes at 90 days compared to a standard BP management.

## Trial status

Currently, 8 centers have been opened and 1 additional center is in the process of opening. The first patient was randomized on March 10th 2021. The 190th patient was randomized on January 18, 2022. This article is based on the version 3.1 of the protocol (14/10/2021).

## Supplementary Information

**Additional file 1.** List of DETERMINE investigators.

**Additional file 2.** Norepinephrine dosage for the experimental arm.

**Additional file 3.** Sedation protocol.

**Additional file 4.** Statistical Analysis Plan.

**Additional file 5.** Original ethic approval document.

## Data Availability

At the end of the trial, all investigators will have access to the data. Data will be made available upon reasonable request, notably for individual patient data meta-analyses.

## Abbreviations

ASPECTS: Alberta stroke program early computed tomography score
ACA: Anterior cerebral artery
BP: Blood pressure
DBP: Diastolic blood pressure
EVT: Endovascular therapy
FO: Functional outcome
ICA: Internal carotid artery
IQR: Interquartile range
IVT: Intravenous thrombolysis
MAP: Mean arterial pressure
MCA: Middle cerebral artery
mTICI: Modified treatment in cerebral infarction score
mRs: Modified Rankin scale
NIHSS: National institutes of health stroke scale
PcomA: Posterior communicating artery
PCA: Posterior cerebral artery
TIA: Transient ischemic attack
SBP: Systolic blood pressure
SD: Standard deviation
