# Supplementary material for: Effect of an individualized versus standard blood pressure management during mechanical thrombectomy for anterior ischemic stroke: the DETERMINE randomized controlled trial
Source: Trials. 2022 Jul 26;23:598. doi: 10.1186/s13063-022-06538-9 (PMC9317065; doi:10.1186/s13063-022-06538-9)
Supplement: Supplementary file 1 — Additional file 1. List of DETERMINE investigators. [file 13063_2022_6538_MOESM1_ESM.docx]

**DETERMINE investigators**

**Hôpital de la Fondation A. de Rothschild**

Benjamin Maïer, Simon Escalard, François Delvoye, Solène Hebert, Hocine Redjem, Stanislas Smajda, Jean-Philippe Desilles, Raphael Blanc, Michel Piotin, Mikael Mazighi, Amélie Yavchitz, Chloé Le Cossec, Ornellia Mophawe, Azedine Djarallah, Perrine Boursin, Laurie-Anne Thion, Abdenour Amarouche, Anoushee Shaffii, Audrey Fogang, Aurore Marcou, Elisabeth Ferri, Fanny Le Garrec, Ines Da Costa, Jean-Marie Moures, Jean-Michel Devys, Malika Omarjee, Marie-Claude Dubois, Marie-Claire Nghe-Mann, Matthieu Dorison, Mélanie Sénéchal, Pascal Le Bigot, Nouria Belhadj-Tahar, Severine Gras, Simon Clariot, Stéphane Merat, Sylvie Froucht-Hirsch, Yasmine Ait Yahia, Pierre Seners, Candice Sabben.

**Hôpital Foch**

Bertrand Lapergue, Morgan Le Guen, Julien Rousset

**Centre Hospitalier Universitaire Toulouse**

Thomas Geeraerts, Christophe Cognard, Jean-Marc Olivot, Lionel Calviere, Maxime Pommier, Edouard Naboulsi

**Centre Hospitalier Universitaire Clermont-Ferrand**

Marc Begard, Camille Boissy, Thibaud Cammas, Bernard Cosserant, Romain Grobost, Adrien Guyot, Katia Levrier, Pierre-Antoine Pioche, Ricardo Moreno, Abderahim Zerroug, Elie Lteif, Emmanuel Chabert, Anna Ferrier, Aurélie Masgrau

**Centre Hospitalier Universitaire Lyon (Hospices Civils de Lyon)**

Baptiste Balanca, Lionel Bapteste, Baptiste Bouchier, Claudio Di Roio, Charles-Antoine Lak, Anisoara Gemanar, Romain Carrillon, Carole Bodonian

**Centre Hospitalier Universitaire de Nancy**

Benjamin Gory, Serge Bracard, René Anxionnat, Marc Braun, Anne-Laure Derelle, Liang Liao, François Zhu, Emmanuelle Schmitt, Sophie Planel, Sébastien Richard, Lisa Humbertjean, Gioia Mione, Jean-Christophe Lacour, Marian Douarinou, Gérard Audibert, Marcela Voicu, Lionel Alb, Marie Reitter, Madalina Brezeanu, Agnès Masson, Adriana Tabarna, Iona Podar, Pauline Bourst, Valérie Georges, Sarah Guy, Fatiha Bechiri.

**Centre Hospitalier Universitaire de Lille**

Pr Benoît Tavernier, Dr Gabriela Julean, Dr Pierre Boussemart, Dr Sidi Hamza Roudies, Dr Dominique Envain, Dr Pierre Appourchaux, Dr Julien Martin, Dr Victor Lestrade, Dr Lucie Della Schiava, Dr Nicolas Bricout, Erine Prévost, Julie Bellet

**Hôpital Lariboisière**

Etienne Gayat, Mikael Mazighi

**Hôpital Pitié Salpétrière**

Vincent Degos, Dupont Julie, Frédéric Clarençon
