# Supplementary material for: Effect of an individualized versus standard blood pressure management during mechanical thrombectomy for anterior ischemic stroke: the DETERMINE randomized controlled trial
Source: Trials. 2022 Jul 26;23:598. doi: 10.1186/s13063-022-06538-9 (PMC9317065; doi:10.1186/s13063-022-06538-9)
Supplement: Supplementary file 2 — Additional file 2. Norepinephrine dosage for the experimental arm. [file 13063_2022_6538_MOESM2_ESM.docx]

**Additionale file 2. Modality for diluted norepinephrine (10 μg/ml) administration in the experimental group**

|  | **Delivery rate (ml/h)** | | | | | | | | | | |
| --- | --- | --- | --- | --- | --- | --- | --- | --- | --- | --- | --- |
| **Norepinephrine (10 μg/ml)** | **weight (kg)** | | | | | | | | | | |
| **μg/kg/min** | 50 | 55 | 60 | 65 | 70 | 75 | 80 | 85 | 90 | 95 | 100 |
| **0.02** | 6 | 6.6 | 7.2 | 7.8 | 8.4 | 9 | 9.6 | 10.2 | 10.8 | 11.4 | 12 |
| **0.03** | 9 | 9.9 | 10.8 | 11.7 | 12.6 | 13.5 | 14.4 | 15.3 | 16.2 | 17.1 | 18 |
| **0.04** | 12 | 13.2 | 14.4 | 15.6 | 16.8 | 18 | 19.2 | 20.4 | 21.6 | 22.8 | 24 |
| **0.05** | 15 | 16.5 | 18 | 19.5 | 21 | 22.5 | 24 | 25.5 | 27 | 28.5 | 30 |
| **0.06** | 18 | 19.8 | 21.6 | 23.4 | 25.2 | 27 | 28.8 | 30.6 | 32.4 | 34.2 | 36 |
| **0.07** | 21 | 23.1 | 25.2 | 27.3 | 29.4 | 31.5 | 33.6 | 35.7 | 37.8 | 39.9 | 42 |
| **0.08** | 24 | 26.4 | 28.8 | 31.2 | 33.6 | 36 | 38.4 | 40.8 | 43.6 | 45.6 | 48 |
| **0.09** | 27 | 29.7 | 32.4 | 35.1 | 37.8 | 40.5 | 43.2 | 45.9 | 48.6 | 51.3 | 54 |
| **0.10** | 31 | 33 | 36 | 39 | 42 | 45 | 48 | 51 | 54 | 57 | 60 |
