# Supplementary material for: Effect of an individualized versus standard blood pressure management during mechanical thrombectomy for anterior ischemic stroke: the DETERMINE randomized controlled trial
Source: Trials. 2022 Jul 26;23:598. doi: 10.1186/s13063-022-06538-9 (PMC9317065; doi:10.1186/s13063-022-06538-9)
Supplement: Supplementary file 3 — Additional file 3. Sedation protocol. [file 13063_2022_6538_MOESM3_ESM.docx]

**Additionale File 3. Sedation protocol**

The choice of sedation (conscious sedation or general anesthesia) will be discussed in a collegial manner between the neurologist, the interventional neuroradiologist and the anesthesiologist, as it is commonly done.

- **Conscious sedation:** This will consist of one of the 3 strategies below:

- Bolus administration of midazolam (0.5 to 1 mg per bolus),

- Propofol infusion (1.0 to 2.0 mg/kg/hr or maintenance of a brain tissue target controlled infusion between 1.0 to 3.0 µg/mL)

- Remifentanil infusion (with a maintenance of a brain tissue target controlled infusion between 0.2 to 2.0 ng/mL).

Doses and combinations of the above drugs will be at the discretion of the anesthesiologist.

Patients under conscious sedation will maintain spontaneous ventilation, receive oxygen therapy, but will not be intubated. The degree of consciousness, pain level and respiratory rate will be measured during the procedure.

**- General anesthesia:**

- **Induction:** given the emergency setting and the risk of inhalation, the induction of general anesthesia will be performed in a rapid sequence by:

- Propofol (bolus of 1.0 to 5.0 mg/kg) or Etomidate (bolus of 0.3 mg/kg) or Ketamine (bolus of 1.0 to 2.0 mg/kg)

- Suxamethonium (bolus of 1.0 mg/kg) or Rocuronium (bolus of 1.2 mg/kg)

- Sufentanil (bolus 0.2 to 0.4 µg/kg) or Remifentanil (0.5 to 1 µg/kg).

Doses and combinations of the above drugs will be at the discretion of the anesthesiologist.

**- Maintenance of general anesthesia:**

- based on body weight:

o Propofol infusion (2.0 to 10 mg/kg/hr)

o And Remifentanil infusion (0.2 to 1.0 µg/kg/min)

- OR with intravenous goal-directed anesthesia

o Propofol with maintenance of a brain tissue target controlled infusion between 2.0 and 6.0 µg/mL

o And Remifentanil with maintenance of a brain tissue target controlled infusion between 1.0 and 5.0 ng/mL

Doses and combinations between the above drugs will be at the discretion of the attending anesthesiologist.

**-Ventilation:**

Ventilatory parameters will be adjusted to maintain normal capnia (EtCO2 between 32 and 34 mm Hg).
