# Supplementary material for: Effect of an individualized versus standard blood pressure management during mechanical thrombectomy for anterior ischemic stroke: the DETERMINE randomized controlled trial
Source: Trials. 2022 Jul 26;23:598. doi: 10.1186/s13063-022-06538-9 (PMC9317065; doi:10.1186/s13063-022-06538-9)
Supplement: Supplementary file 4 — Additional file 4. Statistical Analysis Plan. [file 13063_2022_6538_MOESM4_ESM.docx]

**DETERMINE Statistical Analysis Plan**

**Chloé Le Cossec, Jessica Guillaume, Amélie Yavchitz and Benjamin Maier.**

**V1.0 March 4th 2022**

**Rationale**

Stroke is the second leading cause of death worldwide, the first cause of acquired disability, and the second of dementia (1). Since 2015, the combination of intravenous thrombolysis and endovascular therapy (EVT) has been the standard of care for patients suffering from an acute ischemic stroke (AIS) due to a large vessel occlusion of the anterior circulation (2). Despite successful reperfusion rates now exceeding 90%, 50% of recanalized patients have poor functional outcomes after an AIS treated by EVT (2). This crucial point highlights that new managements, in addition to recanalization therapies, are needed to improve functional outcomes. Extreme blood pressure (BP) values within the early phase of AIS are widely described as associated with poor functional outcomes (3). Recently, several studies (4-6) have suggested associations between BP variability and hypotension during EVT, with worse functional outcomes at 90 days. We recently showed that any variation during EVT in the mean arterial pressure (MAP) beyond 10% of the MAP measured before EVT was associated with worse functional outcomes at 90 days (7). Interestingly, an individualized BP control strategy has been shown to decrease complication rates in general surgery in high-risk patients (8). Applied to AIS patients treated with EVT, an individualized MAP control could maintain cerebral perfusion pressure before recanalization and improve functional outcomes at 90 days. That said, no randomized clinical trial has ever evaluated the benefit of an individualized BP control during EVT on functional outcomes.

Our hypothesis is that an individualized BP control strategy using intravenous administration of diluted norepinephrine (5 to 10 µg/ml) or nicardipine (1 mg/ml) or urapidil (5 mg/ml) to maintain MAP during EVT within 10% of the first MAP measured in the interventional neuroradiology suite (ie, before EVT) would reduce disability at 90 days..

**Objectives**

Primary objective: to assess the efficacy on favorable functional outcomes at 90 days of an individualized BP management during EVT, by maintaining MAP within 10% of the first MAP measured before EVT, compared to a standard BP management during EVT (SBP within 140-180 mm Hg, DBP<105 mm Hg).

Secondary objectives:

To assess the impact of an individualized BP management strategy during EVT on:

1. Functional outcome at 90 days (shift analysis)
2. Excellent functional outcome at 90 days
3. Early neurological improvement
4. Symptomatic intracranial hemorrhage 24 hours after EVT
5. Incidence of intraparenchymal hematoma type 2, 24 hours after EVT
6. Successful reperfusion at the end of EVT
7. Final infarct volume 24 hours after EVT
8. All-cause mortality at 90 days
9. Frequency and duration of hospital stays
10. Description of per procedural BP in each group

In the experimental group, description of the maintenance of MBP within the target range.

**Outcomes**

Primary endpoint: Favorable functional outcome, defined by a modified Rankin Scale (mRS) between 0 and 2 at 90 days (±15 days), assessed by certified neurologists or research nurses, blinded to the intervention.

Secondary endpoints:

1. Distribution of the mRS at 90 days (shift analysis),
2. Excellent functional outcome, defined by a mRS between 0 and 1 at 90 days,
3. Early neurological improvement, defined by a reduction of 8 points or more on the NIHSS or a NIHSS 0-1 24 hours after EVT,
4. Symptomatic intracranial hemorrhage according to the ECASS III definition (9)
5. Symptomatic intracranial hemorrhage 24 hours after EVT according to the SITSMOST definition (10)
6. Successful reperfusion, defined as a modified Treatment In Cerebral Infarctions 2b-3,
7. Final infarct volume 24 hours after EVT on control cerebral imaging,
8. All-cause mortality at 90 days
9. Frequency and length of hospital stays
   1. Duration of initial hospital stay (days),
   2. Number of re-hospitalizations within 90 days,
   3. Cumulative length of hospitalization during the 90 days follow-up.
10. Description of per procedural BP:
    1. Percentage of patients with at least one per-procedural hypotension, total number of per-procedural hypotension, mean duration of hypotension (hypotension defined as MAP<90% of the reference MAP for the experimental group, SBP<140 mmHg for the control group),
    2. Percentage of patients with at least one per-procedural hypertension, total number of per-procedural hypertension, mean duration of hypertension (hypertension defined as MAP>110% of the reference MAP for the experimental group, SBP>180 and DBP>105 mmHg for the control group),
    3. Percentage of patients with at least one per-procedural bradycardia, total number of per-procedural bradycardia, mean duration of bradycardia (bradycardia defined as heart rate <40/min),
    4. BP variability, defined by the coefficient of variation,
11. In the experimental group, time spent within the experimental target (±10% of the reference MAP) during EVT (in minutes and in % of the total EVT duration).

**Subgroup analyses**

Efficacy as defined for the primary outcome will be analyzed according to the following subgroups: Age (<70 years, ≥ 70 years); Time to management ( <180 minutes, 180-360 minutes, >360 minutes); History of hypertension (yes vs no); NIHSS at inclusion ( <17 vs ≥17); Gender (female vs male); Type of sedation (conscious sedation vs general anesthesia); IV thrombolysis (yes vs no) ; Occlusion site (M1-M2, ICA termination, tandem); Inclusion systolic blood pressure (first measurement in the operating room <140, 140-179, ≥180 mmHg); Inclusion mean blood pressure (first measurement in the angio suit <90, 90-110, >110 mmHg); Center.

**Inclusion criteria**

- Adult patients ($\geq$18 years).
- AIS due to an anterior LVO: first and second segments of the middle cerebral artery, first segment of the anterior cerebral artery, intracranial internal carotid artery, tandem occlusions (association of an extracranial occlusion of the cervical internal carotid artery with a proximal anterior LVO).
- Indication of EVT under CS or GA, within the first 6 hours after symptoms onset according to current guidelines (11, 12), or within 24 hours according to neuroradiological criteria previously published (13, 14, 15).
- Affiliation to a social security scheme.

**Exclusion criteria**

- Contraindication or no indication for EVT
- Intubation or GA induction before randomization
- Intra-hospital AIS due to an anterior LVO or due to a medical or surgical procedure (interventional cardiology, cardiac or vascular surgery), or any post-surgery AIS.
- AIS due to a posterior LVO (vertebral artery, basilar artery or posterior cerebral artery occlusions)
- Significant pre-stroke functional disability (modified Rankin Scale >2 at randomization)
- Contraindication to iodinated contrast products
- Patients benefiting from legal protection measures
- Pregnant or breastfeeding woman

**Secondary exclusion criteria (before randomisation)**

Failure to measure and define the reference MAP within a timeframe compatible with the timely emergency management of the patient: difference >10 mmHg between the first 2 MAP measured non-invasively at 1 minute interval, and a third MAP >10 mm Hg of the mean of the first 2 MAP

**Trial design**

DETERMINE is an academic, multicenter, prospective, randomized (1:1 ratio), open-label, with blinded endpoint assessment clinical trial (PROBE).

**Time path of the analysis and locking of the database**

After the follow-up of 50% of planned inclusions, the database will be cleaned and checked for completeness. Upon completion, the database will be locked and sent to the independent trial statistician who will perform an interim efficacy analysis. The study may be stopped in case of a significant result (see below for the alpha risk threshold to be used in the interim analysis).

After the follow-up of the final patient, the last records of the database will be cleaned and checked for completeness. Upon completion, the database will be locked. The data will be sent to the independent trial statistician who will perform the final analysis. The final results will then be shared for consideration with the steering committee of the trial. After obtaining the final results, a manuscript describing the main results of the trial will be submitted for publication.

**Statistical significance level**

A two-sided formulation will be chosen for all tests. For the analysis of the primary endpoint, the alpha risk thresholds to be used in the intermediate and final analysis are 0.003 and 0.049 respectively to conclude that the new strategy is effective.

For secondary endpoints, p-values will not be calculated; effect sizes will be expressed with their 95% confidence intervals.

**Population of analysis**

Analysis will be realized on the intention-to-treat population. Intention-to-treat population is defined as all randomized patients analyzed according to their randomization arm.

**Missing data**

We will report proportions of missing values for all collected variables. For descriptive analyses, only the crude, non-imputed data will be presented. For the regression analyses, missing data (if any, and if less than 20%) will be imputed using multiple imputation methods.

**Descriptive statistics**

A descriptive analysis of the data will be performed. This analysis will include point estimates, numbers and percentages for categorical variables, mean, standard deviation, median and range for quantitative variables. The normality of the continuous variables will be assessed graphically. A description of the missing data for each variable (number and percentage) will be done.

**Primary endpoint analysis**

The primary endpoint is favorable functional outcome at 90 days, defined as a modified Rankin score (mRS) between 0 and 2 inclusive. The rate of patients with a favorable functional outcome at 90 days will be compared between the 2 groups using a mixed-effects logistic regression model, adjusted for the variables considered in the randomization, namely age (<70 vs. ≥70 years), type of anesthesia (general vs. conscious sedation) NIHSS score at inclusion (<17 vs. ≥17), and IV thrombolysis (yes vs. no) as fixed effects, and center as a random effect. The adjusted Odds ratio (OR) will be calculated from this model.

An interim efficacy analysis will be performed after 50% of inclusions. The alpha risk thresholds to be used in the interim and final analysis are 0.003 and 0.049, respectively.

**Secondary endpoints analyses**

The secondary endpoints corresponding to binary variables (i.e., endpoints 2, 3, 4, 5, 6, 8) will also be analyzed using a mixed-effects logistic regression model, adjusted for the variables considered in the randomization, namely age (<70 vs. ≥70 years), type of anesthesia (general vs. conscious sedation) NIHSS score at inclusion (<17 vs. ≥17), and IV thrombolysis (yes vs. no) as fixed effects, and center as a random effect.

The ordinal analysis of the modified Rankin score at 3 months (i.e. criterion 1) will be performed using a mixed ordinal logistic regression model, adjusted on the variables considered in the randomization, namely age (<70 vs. ≥70 years), type of anesthesia (general vs. conscious sedation) NIHSS score at inclusion (<17 vs. ≥17), and IV thrombolysis (yes vs. no) as fixed effects, and center as a random effect.

The analysis of the number of re-hospitalizations within 3 months (i.e. criterion 9b) will be performed using a mixed effects Poisson regression (i.e. quasi Poisson or negative binomial), adjusted on the variables considered in the randomization, namely age (<70 vs. ≥70 years), type of anesthesia (general vs. conscious sedation), NIHSS score at inclusion (<17 vs. ≥17), and IV thrombolysis (yes vs. no) as fixed effects, and center as a random effect.

Secondary endpoints corresponding to quantitative variables (i.e. endpoints 7, 9a, 9c) will be analyzed using mixed-effects linear models, adjusted for the variables considered in the randomization, namely age (<70 vs. ≥70 years), type of anesthesia (general vs. conscious sedation), NIHSS score at inclusion (<17 vs. ≥17), and IV thrombolysis (yes vs. no) as fixed effects, and center as a random effect. In case of skewed distribution of these parameters, lognormal or gamma regression can be used.

Hemodynamic variations (i.e. criterion 10) during EVT and the fraction of operative time spent in the BP target (±10% of the first MAP measured in the experimental group, criterion 11) will be described as detailed in the descriptive statistics section.

Subgroup analyses will be performed in the same manner as for the analysis of the primary endpoint.

**REFERENCES**

1. Collaborators GBD. Global, regional, and national incidence, prevalence, and years lived with disability for 328 diseases and injuries for 195 countries, 1990-2016: A systematic analysis for the global burden of disease study 2016. Lancet. 2017;390:1211-1259

2. Fery-Lemonnier E. La prévention et la prise en charge des accidents vasculaires cérébraux en france. Haute Autorité de Santé. 2006

3. Baron JC. Mapping the ischaemic penumbra with pet: Implications for acute stroke treatment. Cerebrovasc Dis. 1999;9:193-201

4. Smith WS, Lev MH, English JD, et al. Significance of large vessel intracranial occlusion causing acute ischemic stroke and tia. Stroke. 2009;40:3834-3840

5. Powers WJ, Rabinstein AA, Ackerson T, et al. 2018 guidelines for the early management of patients with acute ischemic stroke: A guideline for healthcare professionals from the american heart association/american stroke association. Stroke. 2018;49:e46-e110

6. Turc G, Bhogal P, Fischer U, et al. European stroke organisation (eso) - european society for minimally invasive neurological therapy (esmint) guidelines on mechanical thrombectomy in acute ischemic stroke. J Neurointerv Surg. 2019

7. Lapergue B, Blanc R, Gory B, et al. Effect of endovascular contact aspiration vs stent retriever on revascularization in patients with acute ischemic stroke and large vessel occlusion: The aster randomized clinical trial. JAMA. 2017;318:443-452

8. Goyal M, Menon BK, van Zwam WH, et al. Endovascular thrombectomy after large-vessel ischaemic stroke: A meta-analysis of individual patient data from five randomised trials. Lancet. 2016;387:1723-1731

9. Hacke W, Kaste M, Bluhmki E, Brozman M, Davalos A, Guidetti D, et al. Thrombolysis with alteplase 3 to 4.5 hours after acute ischemic stroke. N Engl J Med. 2008;359(13):1317-29.

10. Wahlgren N, Ahmed N, Davalos A, Ford GA, Grond M, Hacke W, et al. Thrombolysis with alteplase for acute ischaemic stroke in the Safe Implementation of Thrombolysis in Stroke-Monitoring Study (SITS-MOST): an observational study. Lancet. 2007;369(9558):275-82.

11. Powers WJ, Rabinstein AA, Ackerson T, Adeoye OM, Bambakidis NC, Becker K, et al. Guidelines for the Early Management of Patients With Acute Ischemic Stroke: 2019 Update to the 2018 Guidelines for the Early Management of Acute Ischemic Stroke: A Guideline for Healthcare Professionals From the American Heart Association/American Stroke Association. Stroke. 2019;50(12):e344-e418.

12. Turc G, Bhogal P, Fischer U, Khatri P, Lobotesis K, Mazighi M, et al. European Stroke Organisation (ESO) - European Society for Minimally Invasive Neurological Therapy (ESMINT) Guidelines on Mechanical Thrombectomy in Acute Ischemic Stroke. J Neurointerv Surg. 2019.

13. Jovin TG, Chamorro A, Cobo E, et al. Thrombectomy within 8 hours after symptom onset in ischemic stroke. N Engl J Med. 2015;372:2296-2306

14. Nogueira RG, Jadhav AP, Haussen DC, et al. Thrombectomy 6 to 24 hours after stroke with a mismatch between deficit and infarct. N Engl J Med. 2018;378:11-21

15. Albers GW, Marks MP, Kemp S, et al. Thrombectomy for stroke at 6 to 16 hours with selection by perfusion imaging. N Engl J Med. 2018;378:708-718
