# Supplementary material for: Effect of an individualized versus standard blood pressure management during mechanical thrombectomy for anterior ischemic stroke: the DETERMINE randomized controlled trial
Source: Trials. 2022 Jul 26;23:598. doi: 10.1186/s13063-022-06538-9 (PMC9317065; doi:10.1186/s13063-022-06538-9)
Supplement: Supplementary file 5 — Additional file 5. Original ethic approval document. [file 13063_2022_6538_MOESM5_ESM.docx]

M. VACHEY PATRICK

Fondation Ophtalmologique A. de Rothschild /URC

Unité de Recherche Clinique, 29 rue Manin

75019 Paris

Reference CPP Ouest V : 20/053-1 Reference SI CNRIPH : 20.07.01.64648

Internal reference : BMR_2020_19

IDRCB : 2020-A01451-38

Clinical trial type : Category 1 – Non-health product

Subject : Your request to the CPP ouest V for autorisation

Dear Sir,

You have requested the opinion of the CPP Ouest V on the following research project:

***Comparison of an individualized versus standard blood pressure control strategy during mechanical thrombectomy of cerebral infarcts of the anterior circulation (DETERMINE).***

Sponsor : M. VACHEY PATRICK

Coordinating investigator : Dr Benjamin MAÏER

Regarding the provisions of the Public Health Code,

Regarding the documents initially submitted to the committee for the 1st passage of your file in session on Tuesday 08 September 2020

Regarding the additional or amended documents submitted in response to the Committee's requests:

1. Letter of response to the Committee's remarks dated and signed on 14/09/2020,
2. Protocol (version 1.1 of 14/09/2020), with and without apparent changes,
3. Summary (version 1.1 of 14/09/2020), with and without apparent changes,
4. Information notes and consent forms
   - Patient (version 1.1 of 14/09/2020), with and without apparent changes,
   - Patient continuation (version 1.1 of 14/09/2020), with and without apparent changes,
   - Third party (version 1.1 of 14/09/2020), with and without apparent changes,
   - Third party continuation (version 1.1 of 14/09/2020), with and without apparent changes.

Regarding the additional or amended documents submitted in response to the Committee's requests:

1. Letter of response to the Committee's remarks dated and signed 28/09/2020,
2. Information notes and consent forms
   - Patient (version 1.2 of 29/09/2020), with and without apparent changes,
   - Patient continuation (version 1.2 of 29/09/2020), with and without apparent changes,
   - Third party (version 1.2 of 29/09/2020), with and without apparent changes,
   - Third party continuation (version 1.2 of 29/09/2020), with and without apparent changes.

After studying your file and in view of the reports presented at the meeting on Tuesday 8 September 2020, the Committee considered that the conditions of implementation planned were

satisfactory, that the benefit/risk ratio was acceptable and that the information provided to patients was appropriate.

The Comité de Protection des Personnes Ouest V has issued a favourable opinion

to the implementation of your clinical trial.

The following participated in the examination of the dossier at this session:

|  | **Category** | **Licensees** | **Deputies** |
| --- | --- | --- | --- |
| **1^er^ collège** | Biomedical research | M. Jean-Michel REYMANN * | M. Florian NAUDET *  M. Boris CAMPILLO- GIMENEZ *  Mme Marie-Béatrice SAADE * |
|  | General Practitioners | Mme Adeline JOUANNIN * |  |
|  | Hospital pharmacists |  | Mme Claire LAFOREST * |
|  | Nursing staff |  |  |
| **2^ème^ collège** | Qualified persons in the field of  ethics | Mme Annick LE ROL * | M. Fabrice LEDOUX |
|  | Psychologists |  |  |
|  | Social workers |  |  |
|  | Persons qualified in legal matters | M. Jean-Baptiste THIBERT * |  |
|  | Representatives of approved  associations of patients and users of the health system | M. Gérard LE GOFF * |  |

* with voting rights

Yours sincerely.

*Rennes, Tuesday 29th September 2020.*

Jean-Michel REYMANN Président du C.P.P. Ouest V
